# In Vitro Acaricidal Activity of *Atropa belladonna* and Its Components, Scopolamine and Atropine, against *Rhipicephalus* (*Boophilus*) *microplus*

**DOI:** 10.1155/2014/713170

**Published:** 2014-11-12

**Authors:** R. Godara, M. Katoch, R. Katoch, Anish Yadav, S. Parveen, Bhavna Vij, Varun Khajuria, G. Singh, Nirbhay K. Singh

**Affiliations:** ^1^Division of Veterinary Parasitology, Faculty of Veterinary Sciences and Animal Husbandry, Sher-e-Kashmir University of Agricultural Sciences and Technology, R. S. Pura, Jammu 181 102, India; ^2^Indian Institute of Integrative Medicine (CSIR), Canal Road, Jammu 180 001, India; ^3^Department of Veterinary Parasitology, College of Veterinary Science, Guru Angad Dev Veterinary and Animal Sciences University, Ludhiana 141 004, India

## Abstract

In vitro efficacy of methanolic extract of *Atropa belladonna* and its components scopolamine and atropine was assessed against *Rhipicephalus* (*Boophilus*) *microplus*. Five concentrations of the extract (1.25%, 2.5%, 5%, 10%, and 20%) were used whereas scopolamine and atropine were each tested at 0.1%. In adult immersion test, the extract was lethal to ticks at 20% concentration. The LC50 and LC95 values were determined as 6.875% and 17.306%, respectively. The extract caused a significant reduction (*P* < 0.05) in egg mass production at 10% concentration. In larval packet test, the extract was lethal to larvae in the concentrations of 10% and 20% after 24 h, with LC50 and LC95 values of 1.321% and 4.935%, respectively. Scopolamine and atropine showed 93.3% and 60.0% mortality of adult ticks, respectively, but they caused complete (100%) blocking of hatching as well as 100% larval mortality. Scopolamine and atropine were observed to be more potent than the crude extract at an equivalent concentration in both the bioassays.

## 1. Introduction 

*Rhipicephalus* (*Boophilus*)* microplus*, “the tropical cattle tick,” is the major tick species infesting dairy animals in tropical and subtropical regions of the world. In India,* R*. (*B.*)* microplus* is widely distributed and infesting several host species [1]. It causes huge production losses in the form of reduced weight gain and milk production, anaemia, hide damage, and even mortalities [2]; besides it transmits several disease causing pathogens such as* Babesia bovis*,* B. bigemina,* and* Anaplasma marginale* [3]. Economic losses due to tick infestation in livestock of India have estimated as high as US$ 498.7 million per annum [4].

Presently, the tick control is mainly focused on large scale and repeated use of synthetic acaricides. The application of these acaricides has limited efficacy in reducing tick infestation; besides their hazardous effects on environment make their use a concern, mainly because human beings are the indirect target. As a consequence of extensive use of chemicals this tick species has developed resistance to the major classes of acaricides [5, 6] and has stimulated the search for new control strategies. To minimise the use of chemical acaricides, other alternative approaches involve the use of ecofriendly sustainable methods in a strategic integrated manner; along with the use of hosts with natural resistance to ticks and tick vaccines, the exploration of the possibilities of the plant derivatives for the control of ticks has drawn our attention in the last few decades [7, 8]. These phytochemicals act in different ways such as counteraction of growth regulatory hormones, inhibition of egg development, disruption of mating and sexual communication, and inhibition of chitin formation and their repellent action [9]. The efficacy of a single ectoparasiticidal/repellent plant product can be enhanced by a judicious combination with another plant or active ingredient that has adjuvant properties [7].

*Atropa belladonna* (family: Solanaceae), commonly known as “deadly nightshade” or “devil's berries,” is native to Europe, North Africa, and Western Asia. The species can be cultivated in shades of trees, on wooded hills, and on chalk or limestone [10]. It is a perennial herb, reaching about 4.9 ft in height with long ovate leaves. It is hallowed by long tradition as one of the classic poisons of antiquity.* Atropa belladonna* is a rich source of flavonoids, coumarin, atropa-amine, scopolamine, and hyoscyamine [11, 12]. It has long been used in human medicine for an assortment of conditions including headache, menstrual symptoms, peptic ulcer disease, histaminic reaction, inflammation, and motion sickness and as a sedative as well as in homeopathic drugs [12–15]. However, to the best of our knowledge, no study was conducted to investigate the acaricidal activity of* A. belladonna* extract and its active components, scopolamine and atropine. The present study was therefore designed to evaluate the in vitro acaricidal effect of methanolic extract obtained from the aerial parts of* A. belladonna* and its active components, scopolamine and atropine, on* R.* (*B.*)* microplus* of cattle at Jammu, India.

## 2. Materials and Methods

### 2.1. Plant Material and Extraction

Aerial parts of* A. belladonna* were collected from nursery developed near Baba Rishi Shrine Gulmarg, Jammu and Kashmir, India. All parts were cleaned with distilled water and air dried under shade at a well-ventilated place. The plant material was pulverized to powder form with a mixer grinder. Fifty grams of powder were soaked in 250 mL of methanol solvent to exhaustion (for about 16 h). The removal of the solvent at below 40°C temperature, under reduced pressure, and at a rotation speed of 20 rpm in vacuum rotary evaporator yielded the extract. The extract was scrapped off, transferred to an air-tight container, and stored in a freezer at −20°C till subsequent uses.

### 2.2. HPLC Analysis

The extracts obtained from plant sample were prepared in HPLC-grade methanol for quantitative analysis. Standard scopolamine (0.6 mg/5 mL) and atropine (1.0 mg/5 mL) were prepared in HPLC-grade methanol. Analysis was performed with a Shimadzu (Nexera) HPLC system equipped with LC-30AD Liquid Chromatograph, SIL-30AC Autosampler, SPD-M20A Detector, CBM-20A Communication Bus Module, CTO-20AC Column Oven and Lab solutions software. All samples were filtered through 0.45 *μ*M (Millipore, Bedford, MA) filter. Extracts were separated on a RP-18 (4 × 250 mm, 5 *μ*m; Merck, Bangalore, India) column. The mobile phase consisted of 0.05 M KH_2_PO_4_ in water (A) and acetonitrile (B) delivered at a flow rate of 1.0 mL/min. The gradient system was as follows: at 0 minutes 10% B, at 20 minutes 60% B, at 23 minutes 60% B, and at 25 minutes 10% B. The samples were analyzed at 30°C to provide efficiency to the peaks and the UV chromatograms were recorded at 210 nm.

### 2.3. Preparation of Stock and Test Concentrations

The methanolic extract of* A. belladonna* was dissolved in dimethylsulphoxide (DMSO) to prepare stock solution. Serial dilutions of the extract used in the adult immersion test (AIT) and larval packet test (LPT) were made in distilled water, in order to obtain the concentrations of 1.25%, 2.5%, 5%, 10%, and 20%. Scopolamine and atropine were used in the concentration of 0.1% by dissolving them in DMSO.

### 2.4. *Rhipicephalus* (*Boophilus*) microplus Ticks for Bioassays

The dropped engorged-adult female ticks were collected from the cattle sheds located at R.S. Pura, Jammu, Jammu and Kashmir state of India, and brought to the laboratory in wide mouthed glass jars sealed with muslin cloth. The ticks were thoroughly washed with tap water and dried on filter paper towel. The identification of ticks was made under stereomicroscope according to keys and descriptions given elsewhere [3, 16]. These ticks were used in the AIT within 24 h of collection or were incubated at a temperature of 27 ± 2°C and relative humidity of 80 ± 5% (approximately 4 weeks) to collect larvae for LPT.

### 2.5. Adult Immersion Test

The AIT was performed as described by Sharma et al. [17] with minor modifications. The ticks were weighed and assigned to groups randomly (5 ticks per group). The different groups of ticks were immersed in 10 mL of the respective concentrations (1.25%, 2.5%, 5%, 10%, and 20%) of* A. belladonna* and scopolamine (0.1%) and atropine (0.1%) by placing them directly into containers and stirred with glass rod before and after adding ticks. After 5 min, the acaricide was poured off through a sieve and the ticks were transferred to the tissue paper towel for drying and kept separately in glass tubes and sealed with muslin cloth. For each concentration six replications were maintained. Simultaneously, the ticks in the control group were treated with 10% DMSO and six replications were maintained. The treated ticks were kept in desiccator which was kept in BOD incubator at a temperature of 27 ± 2°C and relative humidity of 80 ± 5% for oviposition. The mortality was observed on day 14 posttreatment (PT). The ticks which did not oviposit even after 14 days were considered as dead [17]. The eggs laid by these ticks were collected, weighed, and observed separately at the same condition of incubation for the next 30 days for visual estimation of hatching rate [8].

The reproductive index (RI) and the percentage inhibition of oviposition (IO) were calculated as follows:

(1) $$\begin{array}{c} \text{RI}=\frac{\text{Average}\;\text{weight}\;\text{of}\;\text{eggs}\;\text{laid}\;\;\left(\text{mg}\right)}{\text{Average}\;\text{weight}\;\text{of}\;\text{live}\;\text{ticks}\;\;\left(\text{mg}\right)},\\ \text{IO}\left(\%\right)=\frac{\text{RI}\;\text{of}\;\text{control}\;\text{ticks}-\text{RI}\;\text{of}\;\text{treated}\;\text{ticks}\;}{\text{RI}\;\text{of}\;\text{control}\;\text{ticks}}\times 100. \end{array}$$

### 2.6. Larval Packet Test

In LPT, two-week-old* R*. (*B.*)* microplus* larvae were exposed to different concentrations (1.25%, 2.5%, 5%, 10%, and 20%) of* A. belladonna* and scopolamine (0.1%) and atropine (0.1%) according to FAO [18]. The filter papers (7.5 × 9.0 cm, Whatman No. 1, Maidstone, UK) were immersed in the test concentrations and were dried by keeping the filter papers for at least 1 h in incubator at 37°C. The treated papers were folded in half and sealed on the sides using clamps. About 100 14-day-old larvae were dropped into impregnated filter packets before finally sealing the packet with clamp at the top. The packets were than incubated at a temperature of 27 ± 2°C and relative humidity of 80 ± 5% and subsequent mortality of larvae was quantified after 24 h. Each testing dose was tested six times. The larvae in the control group were treated with 10% DMSO and six replications were maintained.

The mortality rate was obtained according to the following formula [8]:

(2) $$\begin{array}{c} \text{Mortality}\left(\%\right)=\frac{\text{dead}\;\text{larvae}}{\text{total}\;\text{of}\;\text{larvae}}\times 100. \end{array}$$

### 2.7. Statistical Analyses

The significance of the data was calculated by one-way ANOVA test and Duncan's test was used for post hoc analysis. A value of *P* < 0.05 was considered significant. The lethal concentrations (LC_50_ and LC_95_) and their respective 95% confidence intervals (CI) were determined by applying regression equation analysis to the probit transformed data of mortality. The dose-response data were analysed by probit method [19] using Graph Pad Prism 4 software.

## 3. Results

**Figure 1:**
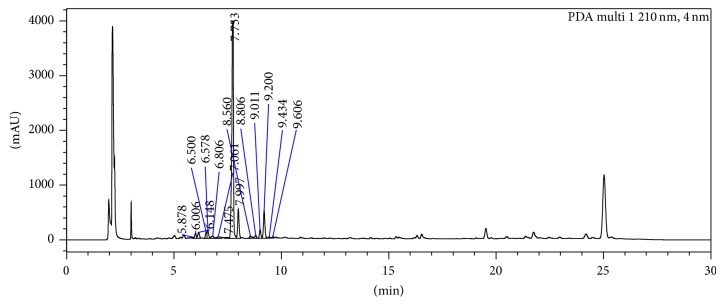
HPLC chromatogram (210 nm) of the* A. belladonna* methanolic extract showing peaks of atropine and scopolamine.

The HPLC analysis revealed the reproducible separations. The HPLC chromatogram at 210 nm showed the peaks of two standard marker compounds scopolamine and atropine at the retention time of 7.06 min and 8.5 min, respectively, in the standard preparation and both the compounds in the* A. belladonna* extract (Figure 1). Resolution of both the marker compounds (scopolamine and atropine) was clear and the marked differences in their retention times made their quantification easier. The* A. belladonna* extract was found to contain 8.84% scopolamine and 2.065% atropine.

**Figure 2:**
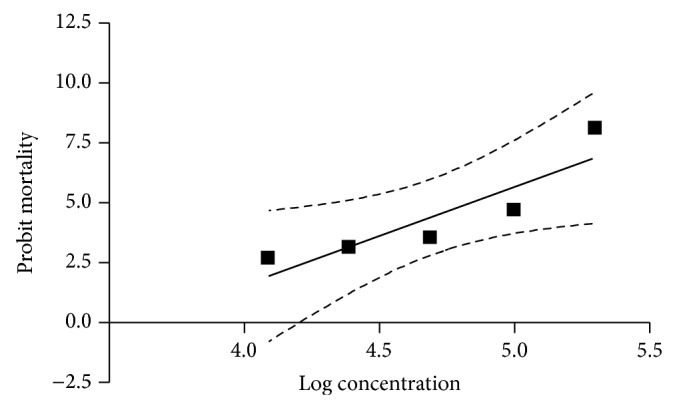
Regression line of probit mortality of* R.* (*B.*)* microplus* against the log concentrations of the extract of* Atropa belladonna* using adult immersion test.

The results on accumulative percentage of mortality for each test with adults are presented in Table 1. The highest mortality (100%) was observed at the concentration of 20%. There were significant differences (*P* < 0.05) between the concentrations used. The regression graph of mean mortality of adult ticks plotted against the values of progressively increasing concentrations of the extract is shown in Figure 2. The regression equation derived after comparing probit mortality versus log values of concentrations of the extract (*y* = 4.0913*x* − 14.791, *R* ^2^ = 0.8049) revealed that 80.49% of correlation with log concentration in probit mortality could be assigned to the concentration of the extract. Based on regression equation the LC_50_ (CI) and LC_95_ (CI) values were calculated as 6.875% (6.615–7.155) and 17.306% (16.024–18.691), respectively (Table 2). The mortality slope value and *R* ^2^ value of the extract determined in the AIT are shown in Table 2. Scopolamine and atropine when used in the concentration of 0.1% caused 93.3% and 60.0% mortality of adult ticks, respectively.

The egg mass laid by the surviving ticks at all the concentrations had lost their glossy appearance. When the IO of treated ticks was compared with the control ticks, it was observed that the ticks treated with 10% concentration of the extract had a reduction of 44.2% and the difference was statistically significant (*P* < 0.05) (Table 1). The ticks treated with 0.1% concentration of scopolamine and atropine showed 60.9% and 54.1% reduction in the oviposition, respectively. The IO slope value and *R* ^2^ value of the extract determined in the AIT are shown in Table 2.

The hatching was completely inhibited at 10% concentration of the extract while the concentrations of 1.25%, 2.5%, and 5% showed 91%, 93%, and 97% inhibition, respectively. Scopolamine and atropine showed 100% inhibition in the concentration of 0.1%.

**Figure 3:**
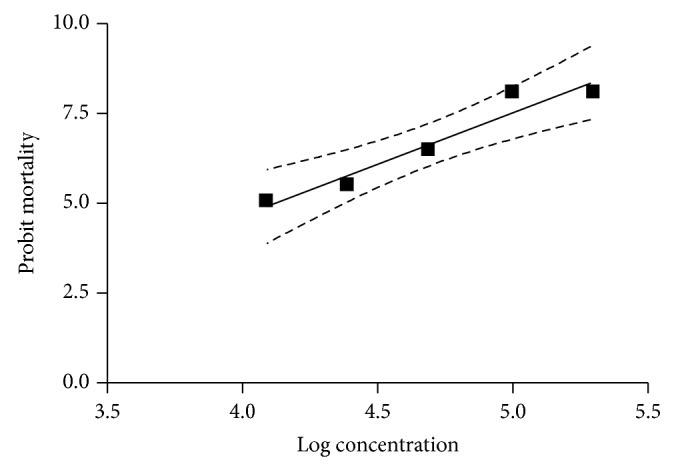
Regression line of probit mortality of* R.* (*B.*)* microplus* against the log concentrations of the extract of* Atropa belladonna* using larval packet test.

In the LPT, the extract caused 100% mortality of larvae at the concentrations of 10% and 20% after 24 h while in the concentrations of 1.25%, 2.5%, and 5%, the mortality rates were found to be 51.7%, 68.7%, and 93.0%, respectively. In the control group, the survival rate of larvae was 100%. There were significant differences (*P* < 0.05) between the concentrations used. The regression graph of mean mortality of larvae plotted against the values of progressively increasing concentrations of the extract is shown in Figure 3. The regression equation derived after comparing probit mortality versus log values of concentrations of the extract (*y* = 2.8645*x* − 6.804, *R* ^2^ = 0.9349) revealed that 93.49% of correlation with log concentration in probit mortality could be assigned to the concentration of the extract. From the regression equation the LC_50_ (CI) and LC_95_ (CI) values were determined to be 1.321% (1.245–1.399) and 4.935% (3.902–6.254), respectively (Table 2). The mortality slope value of the extract determined in the LPT is shown in Table 2. Scopolamine and atropine were lethal to the larvae in the concentration of 0.1%.

## 4. Discussion

The plant extracts or essential oils have long history to control microorganisms and different disease conditions. They have been used for a long time by practitioners of traditional medicine on account of their pharmacological activity and low toxicity. In the recent past various studies have been carried out to develop environmentally safe control measures against ectoparasites and attempts have been made to identify insecticidal/acaricidal properties of different botanical compounds against lice [20, 21], mosquitoes [22], flies [23], ticks [7, 8], and mites [24]. However, in order to offer a complete ecofriendly acaricide, there is a need to assess botanical extracts, especially from plants which are rustic, perennial, and easily cultivable and have a better potential of extension, in addition to their inherent acaricidal properties. The present study establishes that the extract obtained from the aerial parts of* A. belladonna* was toxic to the ticks and the toxicity was directly proportional to the concentration of the extract. The methanolic extract at 20% concentration caused 100% mortality with LC_50_ and LC_95_ values of 6.875 and 17.306%, respectively. On the other hand, when the activities of the individual components (scopolamine and atropine at 0.1% of each) are directly compared with the activities of the extract at an equivalent concentration (corresponding to extract concentration of ~1.25% for scopolamine and ~5% for atropine) both were found to be more potent (Table 1), disregarding the added activity of the other components. Similar results were obtained in the LPT test, where both scopolamine and atropine were lethal at 0.1% concentration, but the crude extract had only 93.0% mortality of larvae of* R.* (*B*.)* microplus* at 5% concentration (containing ~0.44% scopolamine and 0.1% atropine).

In the current study, the effects of* A. belladonna* extract on the future progeny of the exposed ticks were assessed by estimating the inhibition of oviposition. Significant reductions (*P* < 0.05) in the egg mass production in* R.* (*B*.)* microplus* at different levels reflect the direct effect of the extract, because the surviving ticks were weak enough to produce optimum egg mass and thus reflect the population control potentiality of the extract. Further the egg mass laid by the surviving ticks at all the concentrations had lost their glossy appearance that occurs due to waxy water proofing when compared to the eggs laid by control ticks. The role of tick moulting hormone, ecdysteroids on salivary gland function, oogenesis, and oviposition, and its regulation by neurotransmitter dopamine has been investigated in* Amblyomma hebraeum* and* R.* (*B*.)* microplus* [25–28]. It has been reported that, in fully engorged-adult female ticks, the level of ecdysteroids rises in the hemolymph which in turn causes salivary gland to degenerate and as a result ticks lay more eggs. It is therefore strongly speculated that the effect of the extract on hormone and neurotransmitter had caused reductions in the egg mass production in* R.* (*B*.)* microplus*.

In conclusion, the control of ticks presents many great research challenges and prospects for the identification of new, safe, and environmentally acceptable acaricides. The methanolic extract obtained from the aerial parts of* A. belladonna* produced high acaricide activity against larvae and adults of* R.* (*B*.)* microplus*. It also reduced the egg hatchability and the ability to lay eggs of engorged females of* R.* (*B*.)* microplus*. The results of the study suggest it as a potent acaricidal agent for* R. *(*B*.)* microplus*. Further studies on the acaricidal effect of the extract on other tick species, particularly antiovipositional property, make it a valuable component of developing sustainable strategy for integrated tick management. However, in vivo studies are needed to prove that the efficacy is possible without causing the adverse effects on the animal host.

## Figures and Tables

**Table 1:** Tick weight, percent mortality, egg weight, reproductive index (RI), and inhibition of oviposition (IO) of *R*. (*B*.) *microplus* adults exposed to different concentrations of methanolic extract of *Atropa belladonna* and its constituents, scopolamine and atropine.

Conc. (%)	Number of Ticks	Live tick wt (mg) (mean ± SE)	Mortality (%) (mean ± SE)	Egg wt (mg) (mean ± SE)	RI (mean ± SE)	IO (%)
Control	30	104.0 ± 6.4	0.0 ± 0.0^a^	55.6 ± 3.7	0.53 ± 0.01^a^	—
1.25	30	104.7 ± 3.6	0.0 ± 0.0^a^	52.9 ± 3.9	0.50 ± 0.03^a^	5.8 ± 4.3^a^
2.5	30	117.3 ± 11.1	3.3 ± 3.3^a^	54.6 ± 6.7	0.46 ± 0.03^b^	14.5 ± 5.1^a^
5.0	30	109.6 ± 4.6	6.7 ± 3.3^a^	43.3 ± 4.7	0.39 ± 0.04^b^	26.4 ± 6.2^a^
10.0	30	108.1 ± 4.8	36.7 ± 8.8^b^	29.8 ± 2.7	0.30 ± 0.03^c^	44.2 ± 5.7^b^
20.0	30	103.2 ± 4.2	100.0 ± 0.0^c^	0.0 ± 0.0	0.0 ± 0.0^d^	100.0 ± 0.0^c^
Scopolamine (0.1%)	30	115.0 ± 7.5	93.3 ± 6.7^c^	32.3 ± 4.8	0.21 ± 0.03^d^	60.9 ± 5.0^c^
Atropine (0.1%)	30	130.7 ± 6.2	60.0 ± 11.5^d^	55.8 ± 5.7	0.25 ± 0.04^e^	54.1 ± 7.7^d^

Mean followed by the same letters in the same column does not differ statistically at a significance level of 5%.

**Table 2:** Dose-response data of *R*. (*B*.) *microplus* against methanolic extract of *Atropa belladonna* using adult immersion test (AIT) and larval packet test (LPT).

Test	Variables	Slope ± SE	*R* ^2^	LC_50_ (%) (95% CI)	LC_95_ (%) (95% CI)
AIT	Mortality	4.091 ± 1.16	0.8049	6.875 (6.615–7.155)	17.306 (16.024–18.691)
	Egg mass	−43.14 ± 10.62	0.8461		
	RI	−0.3830 ± 0.09	0.8458		
	% IO	72.00 ± 17.52	0.8491		
LPT	Mortality	2.865 ± 0.44	0.9349	1.321 (1.245–1.399)	4.935 (3.902–6.254)

RI: reproductive index; IO: inhibition of oviposition; CI: confidence interval.
